# Metabolic and Immunological Shifts during Mid-to-Late Gestation Influence Maternal Blood Methylation of *CPT1A* and *SREBF1*

**DOI:** 10.3390/ijms20051066

**Published:** 2019-03-01

**Authors:** Shilpa Pavethynath, Chihiro Imai, Xin Jin, Naomi Hichiwa, Hidemi Takimoto, Motoko Okamitsu, Iori Tarui, Tomoko Aoyama, Satoshi Yago, Ayako Fudono, Masaaki Muramatsu, Naoyuki Miyasaka, Noriko Sato

**Affiliations:** 1 Department of Molecular Epidemiology, Medical Research Institute, Tokyo Medical and Dental University, Tokyo 113-8510, Japan; shilpa.epi@mri.tmd.ac.jp (S.P.); imai.epi@mri.tmd.ac.jp (C.I.); ma174004@tmd.ac.jp (X.J.); ma170073@tmd.ac.jp (N.H.); muramatsu.epi@mri.tmd.ac.jp (M.M.); 2 Department of Nutritional Epidemiology, National Institute of Health and Nutrition, Tokyo 162-8636, Japan; thidemi@nibiohn.go.jp (H.T.); nhtun@nibiohn.go.jp (I.T.); tomokom@nibiohn.go.jp (T.A.); 3 Child and Family Nursing, Graduate School of Health Care Sciences, Tokyo Medical and Dental University, Tokyo 113-8510, Japan; motoko.cfn@tmd.ac.jp (M.O.); sycfn@tmd.ac.jp (S.Y.); 4 Comprehensive Reproductive Medicine, Graduate School of Medical and Dental Sciences, Tokyo Medical and Dental University, Tokyo 113-8510, Japan; faya.per@tmd.ac.jp (A.F.); n.miyasaka.gyne@tmd.ac.jp (N.M.)

**Keywords:** pregnancy, DNA methylation, *CPT1A*, *SREBF1*, leukocyte composition

## Abstract

Mid-to-late gestation is a unique period in which women experience dynamic changes in lipid metabolism. Although the recent intensive epigenome-wide association studies (EWAS) using peripheral leukocytes have revealed that lipid-related traits alter DNA methylation, the influence of pregnancy-induced metabolic changes on the methylation levels of these differentially methylated sites is not well known. In this study, we performed a prospective cohort study of pregnant women (*n* = 52) using the MassARRAY EpiTYPER assay and analyzed the methylation levels of variably methylated sites, including *CPT1A* intron 1 and *SREBF1* intron 1 CpGs, which were previously verified to be robustly associated with adiposity traits. Although methylation of *SREBF1* was associated with body mass index (BMI) and low-density lipoprotein cholesterol at mid-gestation, this association was attenuated at late gestation, which was consistent with the metabolic switch from an anabolic to a catabolic state. However, the BMI association with *CPT1A* intron 1 methylation appeared to strengthen at late gestation; this association was mediated by pre-pregnancy BMI-dependent change in the leukocyte proportion during mid-to-late gestation. Thus, the methylation of adiposity-related differentially methylated regions was sensitive to metabolic and immunological changes during mid-to-late gestation.

## 1. Introduction

Pregnancy causes extensive changes in a mother’s body to provide optimal physiological conditions for fetal growth. Changes in maternal metabolism from an anabolic to a catabolic state occur notably from the second to the third trimester, mainly characterized by alteration in lipid metabolism [1,2,3]. Progressive insulin resistance during this period might mediate adaptation in lipid and glucose metabolism to meet the energy demand for fetal growth [2,4]. Appropriate gestational weight gain during this period is also important for fetal growth [5]. Thus, mid-to-late gestation (spanning several months) is a unique period in which women experience dynamic changes in the internal environment of the body. Obesity and pregnancy share some features in terms of adiposity-related changes of the internal environment, but pathological adiposity and gestational weight gain are not the same. It has also been demonstrated in many studies that a high BMI during pregnancy increases the risks for both mothers and children to develop certain diseases [6]. It is important to investigate the underlying epigenetic changes. Epigenetics include the chemical modifications to DNA, which are vulnerable to environmental stimuli and potentially involved in gene regulation. The most common modification, called DNA methylation, is the addition of a methyl group onto the 5′ carbon of cytosine within the DNA sequences of a 5′-cytosine-phosphate-guanine-3′ (CpG) dinucleotide. The identification of CpG sites which are differentially methylated by phenotype alteration is the first step in deciphering the connection between epigenetics and diseases. Although recent epigenome-wide association studies (EWAS) revealed that obesity and lipid-related traits altered peripheral leukocyte DNA methylation at a large number of CpG sites, it is not well known how pregnancy-induced physiological changes influence methylation levels on the adiposity-related methylation sites.

Generally, the adiposity trait is assessed by body weight or body mass index (BMI) and serum lipid levels. Several independent EWAS of BMI [7,8,9,10,11] have consistently identified the same set of BMI-associated CpG sites. Similarly, multiple EWAS of blood lipid traits also identified a common set of lipid-associated CpG sites [12,13,14,15,16,17,18,19,20]. Among those CpGs, cg00574958 and cg17058475 of carnitine palmitoyltransferase 1A gene (*CPT1A*) and cg11024682 of sterol regulatory element-binding protein gene 1 gene (*SREBF1*) repeatedly appeared as top association sites. Importantly, DNA methylation of these CpGs was associated with BMI and gene expression, and gene expression was also associated with BMI [7,10,15]. *CPT1A* encodes a rate-limiting enzyme of fatty acid β-oxidation, involved in the transport of fatty acid from the mitochondrial outer membrane to the inner membrane [21]. The methylation state of the two CpGs in *CPT1A* intron 1 is thought to be indicative of the promoter activity of *CPT1A* [8]. *SREBF1* encodes a lipid metabolism transcriptional factor, promoting the conversion of free fatty acids to triglyceride in the liver and to triglyceride-rich lipoproteins in the blood stream [22]. It has been proposed that epigenetic regulation of *SREBF1* intron1 CpG may have a causal association with BMI; however, it has not yet been determined [7,10]. Furthermore, *CPT1A* has additional variably methylated regions (the representative CpG is cg14249520) in exon 15, which was reported to be associated with prenatal famine exposure [23]. Although the association between *CPT1A* exon 15 methylation and BMI has not been identified in any EWAS reports, only Tobi et al. reported its weak association with serum low-density lipoprotein cholesterol (LDL-C) level [23]. Since these studies have not been conducted in pregnant women, it is not known how pregnancy can influence the methylation levels of these sites.

Here, we performed a prospective cohort study of pregnant women and investigated the association of methylation in *CPT1A* and *SREBF1* with adiposity traits during mid-to-late gestation. Although gestational weight gain does not occur only due to an increase in fat mass [24], we mainly observed the same trend as reported in obesity-related EWAS findings at mid-gestation for the association of those methylation levels with BMI and LDL-C. However, during late gestation, while the association of methylation with adiposity traits was mostly weakened, only *CPT1A* intron 1 methylation showed a significant association with BMI. Unique to late gestation, it was revealed that the lymphocyte proportion was an intermediary step in the causal pathway from BMI (exposure) to the methylation outcome, which is a different situation from usual cases where the lymphocyte proportion can be considered as a covariate (or a confounding factor) (this description uses statistical terms in the context of causal inference). *CPT1A* exon 15 methylation was influenced by leukocyte composition but associated with neither BMI nor LDL-C. In this study, we demonstrate a unique example of a candidate gene methylation analysis by tracing the same individuals and simultaneously assessing the leukocyte composition, which has not always been carefully conducted. With this approach, we have successfully shown that the blood methylation of adiposity-related differentially methylated CpGs during mid-to-late gestation followed metabolic and immunological alteration with advancing gestation. 

## 2. Results

### 2.1. Pregnancy-Induced Adiposity Trait

**Figure 1:**
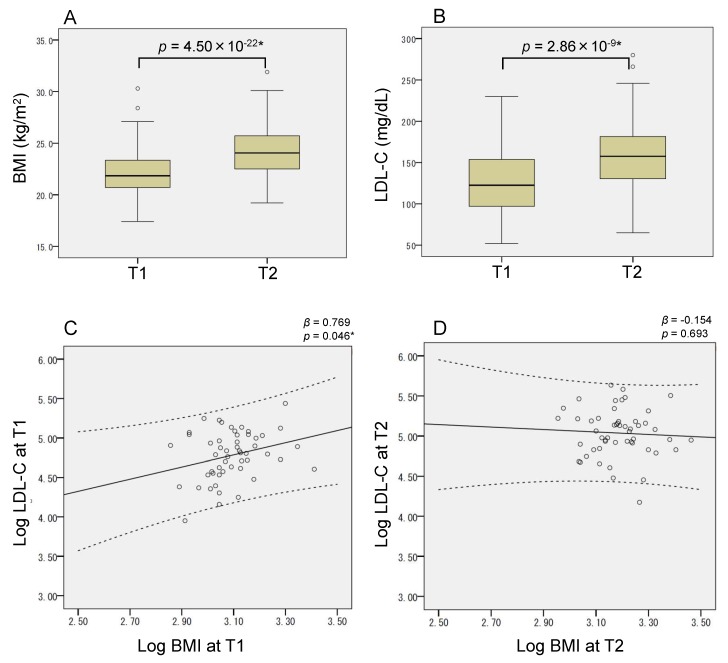
Adiposity traits. (**A**) BMI and (**B**) LDL-C were increased from time point T1 to T2 during mid-to-late gestation. Box and whisker plots illustrate medians, minimum values, maximum values, and interquartile ranges. Scatter plots showing the relationship between BMI and LDL-C at (**C**) T1 and (**D**) T2. (**C**,**D**) Because of the right-skewness, BMI and LDL-C were log transformed. Solid line, a regression line; dotted line, 95% confidence intervals. Statistics were calculated using (**A**,**B**) paired *t*-testing and (**C**,**D**) regression analysis. (BMI, body mass index; LDL-C, low-density lipoprotein cholesterol).

Fifty-two pregnant women with singleton term delivery were analyzed in this study (Section 4.1 and Table S1). The BMI and serum LDL-C of all the women were measured twice, mid-gestation (time point T1, 20.6 ± 4.8 weeks of gestational age) and late gestation (time point T2, 35.7 ± 0.7 weeks). The mean BMI increased from 22.2 ± 2.5 kg/m^2^ (T1) to 24.3 ± 2.7 kg/m^2^ (T2) (*p* = 4.50 × 10^−22^) (Figure 1A). The relationship between pre-pregnancy BMI and gestational BMI (at T1 and T2) was well explained by the quadratic model, which showed that gestational BMI is well correlated with pre-pregnancy BMI. However, the degree of weight gain in people with higher BMI was comparatively lesser (Figure S1). The mean LDL-C levels also increased from 124.6 ± 36.7 mg/dL (T1) to 160.6 ± 46.7 mg/dL (T2) (*p* = 2.86 × 10^−9^) (Figure 1B). Although both BMI and LDL-C increased during mid-to-late gestation, they correlated with each other at T1 but not at T2 (Figure 1C,D).

### 2.2. Mean Methylation Levels of Variably Methylated Sites in CPT1A and SREBF1 during Pregnancy

We analyzed the methylation levels of *CPT1A* intron 1 including cg00574958 (*CPT1A* intron 1 CpG_2) and cg17058475 (*CPT1A* intron 1 CpG_8), *SREBF1* intron 1, and *CPT1A* exon15 regions, in which methylation levels are known to vary among individuals (Table 1 and Table S2). The mean methylation levels of all the CpG sites in *CPT1A* intron 1 were low and did not differ between T1 and T2. The mean methylation levels of *SREBF1* intron 1 were at an intermediate level and the methylation of CpG_4 significantly increased from T1 to T2 to a small extent (1.4% in methylation level). In contrast, the mean methylation levels of CpG sites in *CPT1A* exon 15 were significantly increased from T1 to T2 (2–4% methylation increase). 

### 2.3. Confirmative Study of the Association between Adiposity Phenotype and Methylation Levels

**Figure 2:**
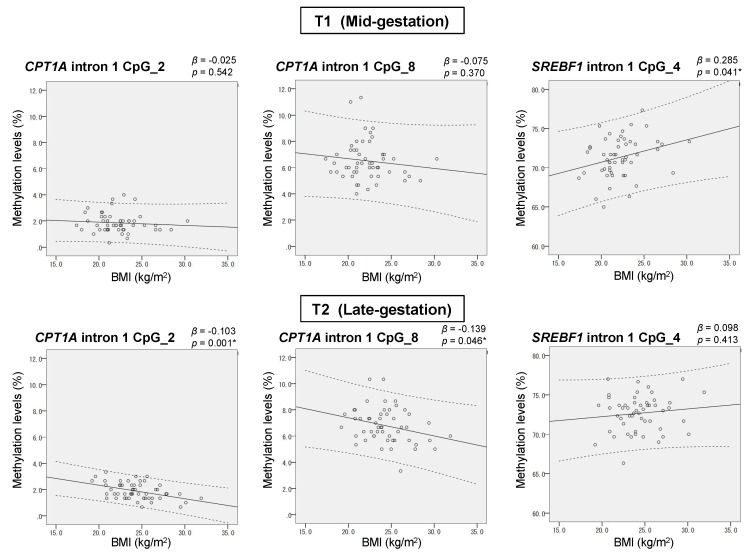
Scatter plots showing the relation between BMI and the methylation levels of adiposity-related methylation sites at two time points during pregnancy. Solid line, a regression line; dotted line, 95% confidence intervals. The scale is adjusted to the methylation for each CpG site. β (regression coefficient) stands for the % change in methylation per unit increase (1 kg/m^2^) in BMI.

For CpG_2 and CpG_8 of *CPT1A* intron 1 and CpG_4 of *SREBF1* intron 1, we analyzed the association of methylation levels with BMI at the two time points T1 and T2 (Figure 2). The direction of association between BMI and the methylation of *CPT1A* was negative, while that of *SREBF1* intron 1 was positive, indicating that we reproduced the association trend identified in EWAS. The magnitude of this association was small (within ±0.3% methylation increase per 1 kg/m^2^ BMI increase), which was also consistent with the previous reports. The association of BMI with methylation levels was significant at T1 for CpG_4 of *SREBF1* (*p* = 0.041) and at T2 for CpG_2 (*p* = 0.001) and CpG_8 (*p* = 0.046) of *CPT1A* intron 1. Next, we analyzed the association between serum LDL-C and methylation levels of the same CpG sites along with the CpG sites in *CPT1A* exon 15 (Figure S2). At mid-gestation (T1), the overall relationship of *CPT1A* and *SREBF1* intron 1 methylations with LDL-C was largely similar to that with BMI. A significant association between *SREBF1* CpG_4 methylation and LDL-C was also detected at T1 (*p* = 0.023). At late gestation (T2), the association of *SREBF1* CpG_4 methylation with adiposity, with regard to both BMI and LDL, was weakened compared to at T1. In contrast, for the association with *CPT1A* intron 1 methylation at T2, we found great discordance between BMI and LDL-C: the methylation was associated with BMI but not with LDL-C (Figure 2 and Figure S2A). Regarding the methylation level of CpG_2.3 of *CPT1A* exon 15, which was previously reported to be positively associated with LDL-C, no association was observed in our study (Figure S2B).

### 2.4. Methylation-Based Estimation of Cell Type Composition

**Figure 3:**
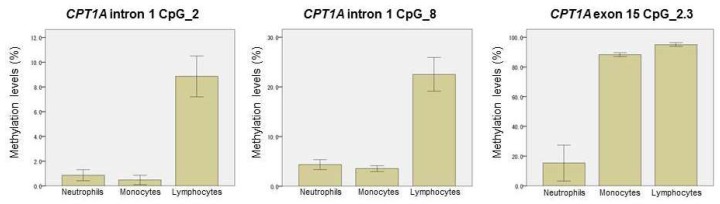
Cell-type-specific methylation levels. Values are given as the mean ± SD. Freshly isolated blood cells from women (control) (*n* = 7) were fractionated using an immunomagnetic negative selection method.

It is known that methylation levels in *CPT1A* intron 1 CpG_2, CpG_8, and *CPT1A* exon 15 CpG_2.3 are cell type dependent [10,25]. Therefore, we separately measured the methylation levels in fractionated blood cells (neutrophils, monocytes, and lymphocytes) derived from the female controls whose age and BMI (age = 31.1 ± 9.4, BMI = 21.2 ± 2.6 kg/m^2^) were similar to those of the pregnant women. Figure 3 shows that the methylation levels of *CPT1A* intron 1 and exon 15 were dependent on the proportions of lymphocytes and neutrophils, respectively. To estimate the individual neutrophil proportion, we first constructed a standard curve showing the relationship between the neutrophil proportion (*x*) and the methylation level (*y*) of the neutrophil methylation marker site (*STK24* intron 1 CpG_2) (Figure S3, Section 4.5). This relationship was approximated by the following formula:

*y* = 0.746*x* + 0.670.

We then measured the methylation levels of the same neutrophil marker for all the pregnant women and then estimated their neutrophil proportion by using the formula

*x* = (*y* − 0.670)/0.746.

**Figure 4:**
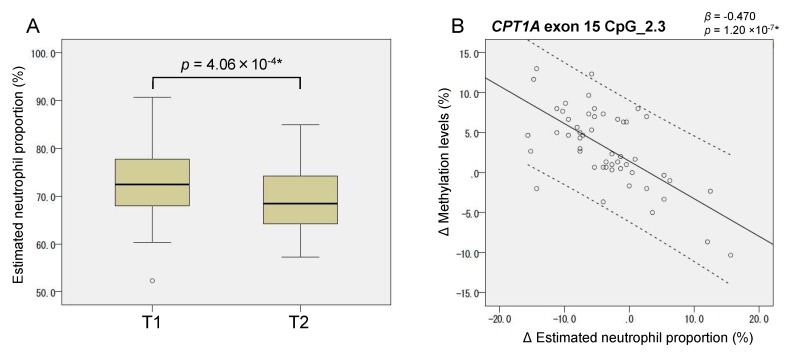
(**A**) Estimated neutrophil proportions at two specific time points during pregnancy. Box and whisker plots illustrate medians, minimum values, maximum values, and interquartile ranges. (**B**) Association of *CPT1A* exon 15 methylation change and neutrophil proportion change from T1 to T2. β indicates methylation change (%) per unit (1%) increase in neutrophil proportion from T1 to T2. Solid, a regression line; dotted, 95% confidence intervals. Statistics were calculated using (**A**) paired *t*-testing and (**B**) regression analysis.

As a result, the mean neutrophil proportion was found to have decreased from T1 to T2 (Figure 4A), which explained the increase in the mean methylation levels of CpG_2.3 of *CPT1A* exon 15 (Table 1). Figure 4B shows that the intra-individual methylation change (∆ methylation) of *CPT1A* exon 15 from T1 to T2 was significantly associated with the intra-individual neutrophil proportion change (∆ neutrophil proportion) during this period (*p* = 1.2 × 10^−7^). We also asked whether we could obtain a similar estimation for neutrophil proportion change (∆ neutrophil proportion) using a different neutrophil-specific marker such as cg17385088. As shown in Figure S4, the correlation between the two estimates using different markers was high (*r* = 0.714). Because the monocyte proportion is relatively small and constant during pregnancy [26], we assumed that the monocyte proportion is invariant and equal to the women’s mean levels (control) (6.5%). Accordingly, the lymphocyte proportions were approximated by subtracting the neutrophil proportion from the total proportions of leukocytes excluding monocytes (93.5—neutrophil proportion) (%).

### 2.5. Dependency of the Lymphocyte Proportion Change on Pre-Pregnancy BMI Influenced the Association between CPT1A Intron1 Methylation and BMI at Late Gestation (T2)

**Figure 5:**
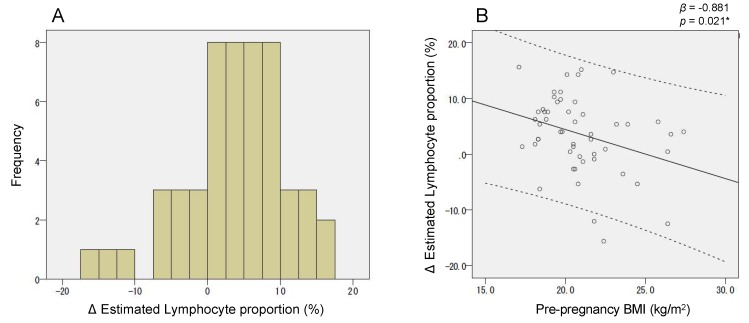
Intra-individual lymphocyte proportion change during mid-to-late gestation was associated with pre-pregnancy BMI. (**A**) Histogram of estimated lymphocyte proportion change (∆ lymphocyte proportion) (%). (**B**) Association between pre-pregnancy BMI and estimated lymphocyte proportion change (∆ lymphocyte proportion).

**Figure 6:**
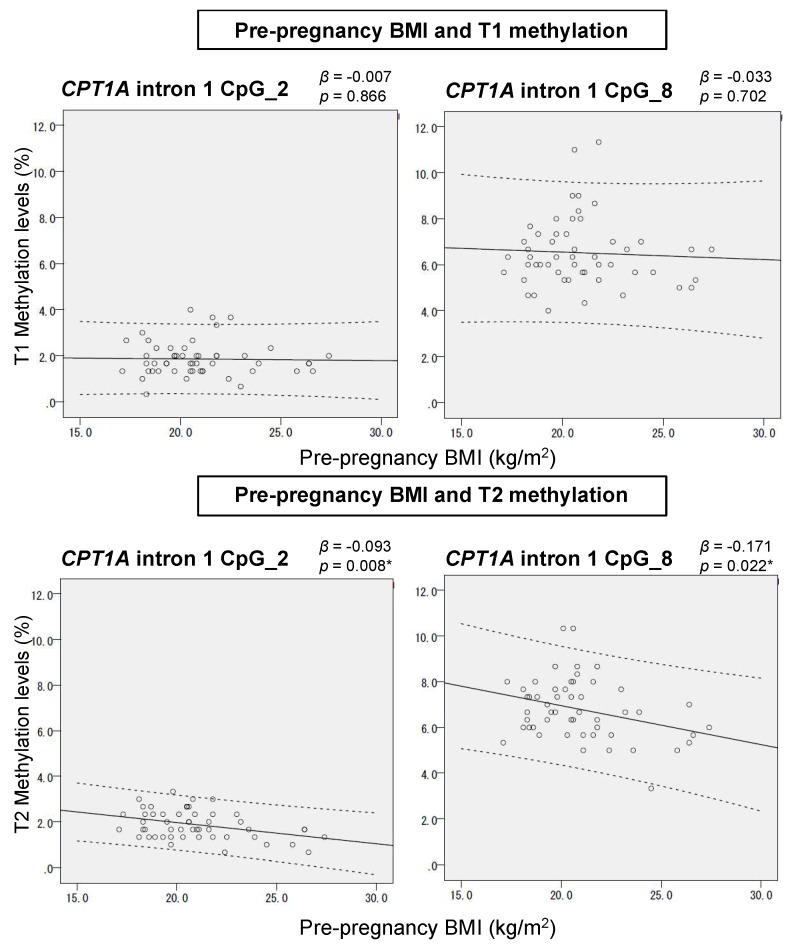
Scatter plots showing the relationship between pre-pregnancy BMI and *CPT1A* intron1 methylation. Solid line, a regression line; dotted line, 95% confidence intervals. The scale is adjusted to the methylation for each CpG site. β (regression coefficient) stands for the % change in methylation per unit increase (1 kg/m^2^) in BMI.

**Figure 7:**
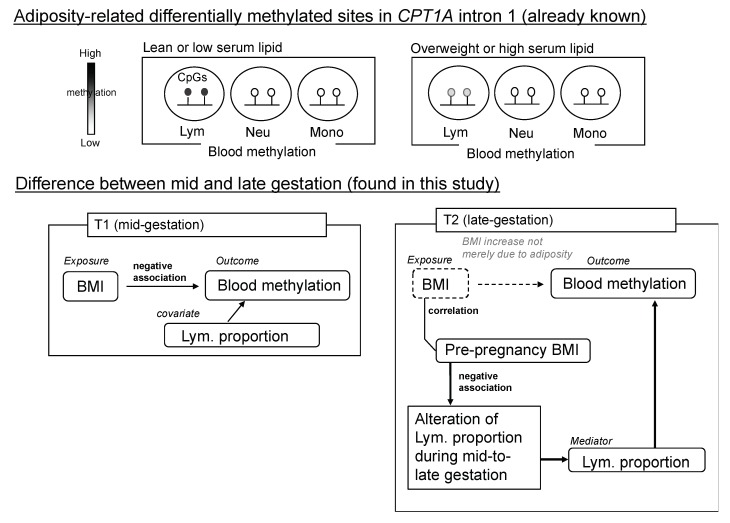
In late gestation, lymphocyte proportion mediates the effect of BMI on blood methylation of *CPT1A* intron 1. Lym, lymphocytes; Neu, neutrophils; Mono, monocytes.

The leukocyte composition was altered during mid-to-late gestation; however, the mode (direction and magnitude) of this alteration varied among individuals. As shown in Figure 5A, there was considerable inter-individual variation in the estimated lymphocyte proportion change (∆ lymphocyte proportion). Importantly, we found that the estimated change (∆ lymphocyte proportion) was negatively associated with pre-pregnancy BMI (*p* = 0.021) (Figure 5B). Meanwhile, *CPT1A* CpG_2 and CpG_8 methylation were positively correlated with lymphocyte proportion (Figure S5). As shown in Figure 6, the relation between pre-pregnancy BMI and *CPT1A* intron1 methylation was different between T1 and T2. Unique to late gestation (T2), BMI was associated with *CPT1A* intron 1 methylation through lymphocyte proportion at T2, which works as an intermediary step in the causal relation from BMI to the methylation outcome (Figure 7).

## 3. Discussion

This study analyzed how pregnancy-induced weight gain or hyperlipidemia influences the methylation levels of obesity-related differentially methylated CpG sites in blood cells. Because mid-to-late gestation is a unique period in which women experience dynamic changes in their internal metabolic environment, investigation of the association between adiposity and the relevant DNA methylation in pregnancy could provide unprecedented information. In this study, we mainly analyzed *CPT1A* intron 1 and *SREBF1* intron 1 methylation, which were already verified to be associated with adiposity traits [7,8,9,10,11,12,13,14,15,16,17,18,19,20]. First, only at mid-gestation, we reproduced the EWAS-identified association between methylation and the two adiposity traits BMI and LDL-C. Second, at late gestation, the overall association of methylation with LDL-C was weakened; this probably reflects the metabolic shift, unique to pregnancy, from an anabolic to a catabolic state. Third, at late gestation, there was discordance between *CPT1A* and *SREBF1* methylation in terms of BMI association. While the association of *SREBF1* methylation with BMI was weakened at late gestation as expected, the association of *CPT1A* with BMI was apparently strengthened. Because *CPT1A* intron 1 methylation depends on the leukocyte composition, we estimated the leukocyte composition by separately measuring the methylation levels of the cell-type-specific marker CpG. Then, we revealed that since pre-pregnancy BMI influenced both the leukocyte composition and BMI at late gestation, the association between BMI and methylation became seemingly strengthened. Thus, our pilot study in a limited number of pregnant women was able to detect a substantial difference in the adiposity-related methylation of *CPT1A* and *SREBF1* between mid and late gestation, which was linked to the metabolic shift and pre-pregnancy-BMI-dependent immunological alteration.

Recently, it was shown that circulating lipids can alter DNA methylation in blood cells [13]. Similarly, the alteration in blood DNA methylation was shown to be predominantly the consequence of adiposity indicated by BMI and not the cause [10]. The mechanisms through which the lipid or adiposity traits change the methylation are currently not clear, but blood-based methylation markers are believed to be important for diagnostic or prognostic purposes. The *CPT1A* intron 1 and *SREBF1* intron 1 CpGs are the representative differentially methylated CpGs associated with both lipid level and BMI. The association of DNA methylation of *CPT1A* intron 1 with blood lipid or metabolic syndrome was identified even in isolated CD4^+^ T cells, indicating that the internal environmental condition in the dysregulated metabolic state may alter *CPT1A* methylation in favor of gene expression in T lymphocytes, independent of cell type composition change [8,17]. Meeks et al. speculated that hypo-methylation at this locus could be the result of high fat mass which requires a more active fatty acid metabolism [12]. Alternatively, it could be considered consistent with the animal experiment that found that CPT-1 inhibition, even being linked to reduced fatty acid oxidation, improved whole-body glucose tolerance and insulin sensitivity [27]. The methylation of *SREBF1* intron 1 was increased by higher blood lipid levels, which was further associated with lower expression of *SREBF1* [7,10,13]. This observation was robust, but not straightforwardly linked to the fact that SREBF1 induces lipogenesis [28]. Mendelson et al. proposed that the methylation level of *SREBF1* intron 1 in the adrenal gland (tissue other than blood) would be involved in the development of obesity and that methylation changes in blood represent a biomarker of trans-tissue differential methylation [7]. Based on these facts, we analyzed the effect of pregnancy-induced adiposity traits (as an exposure) on the methylation of adiposity-related CpGs (as an outcome) in a statistical causal inference framework by using a linear regression model. 

Initially, we analyzed how adiposity traits developed during mid-to-late gestation (Figure 1). There were three and six patients diagnosed with “gestational diabetes mellitus” and “thyroid diseases”, respectively, in our cohort. All the methylation outcomes of those patients were not different with those of the healthy mothers within the same BMI range, regardless of the presence or absence of receiving medication. Therefore, we included data from all the participants in the association analyses in this study. Although both BMI and LDL-C increased during mid-to-late gestation, the correlation between them was only detected at mid-gestation (T1). Consistently, the methylation levels of adiposity-related differentially methylated CpGs did not change much despite the increase in BMI and LDL-C from T1 to T2 (Table 1). In addition, the association of these methylation levels with BMI and LDL-C was mostly reproduced as EWAS report only at mid-gestation (Figure 2 and Figure S2). Weight gain during pregnancy is not merely caused by fatty mass [24]. Considering that the mother is in an anabolic condition during the first two-thirds of gestation to increase her fat depots and that she switches to a catabolic condition during the last one-third of gestation [29], it is reasonable that the overall adiposity associations of the methylation were weakened at T2. This result indicated that the blood methylation of adiposity-related differentially methylated CpGs could follow the internal metabolic changes that occur during advancing gestation.

However, a remarkable exception was found. The BMI association of *CPT1A* intron 1 methylation was unexpectedly strengthened at late gestation. Since it was known that *CPT1A* intron 1 methylation is dependent on the leukocyte composition (Figure 3), we further conducted methylation analysis of the cell-type-specific methylation marker sites. We generated the standard curve to show the relationship between the observed neutrophil proportion and the methylation of the neutrophil-specific marker site (Figure S3) and we estimated the individual neutrophil proportion. The validity of this estimated neutrophil proportion was experimentally supported by the significant association between the neutrophil proportion change (∆) and the methylation change (∆) of *CPT1A* exon 15 (Figure 4). It was consistent with the result that *CPT1A* exon 15 methylation is neutrophil-dependent (Figure 3). Although what determines the leukocyte composition change (∆) during mid-to-late gestation is not known, we found that it is associated with pre-pregnancy BMI (Figure 5). This implied in the statistical model that BMI and leukocyte composition are no longer independent variables to predict methylation at late gestation. In other words, there is a mediation path at late gestation from BMI (exposure) to methylation (outcome) through which the lymphocyte proportion works as a mediator (Figure 7). *CPT1A* intron 1 methylation is positively associated with lymphocyte proportion, and lymphocyte proportion change (∆) is negatively associated with pre-pregnancy BMI (Figure S5 and Figure 5). This relation gave a seemingly strengthened association signal between *CPT1A* intron 1 methylation and BMI at late gestation (Figure 2, Figure 6 and Figure 7). Through this analysis, we also found that there is considerable inter-individual variation in leukocyte composition changes during mid-to-late gestation; this fact has not been paid much attention. Since chronological immune regulation is critical for a successful pregnancy [30], methylation-based leukocyte composition information for pregnant women would be potentially helpful for perinatal care.

A limitation of this study was the small sample size. Thus, larger prospective study cohorts are necessary to validate the results reported here. A strength of this study was that the methylation-based estimation of leukocyte proportion was conducted using controls whose age, sex, and BMI were similar to those of the pregnant women. Most precedential studies of candidate gene methylation (not genome-wide array-based studies) have lacked information about the cell type composition and cell type dependency of methylation. Analyses conducted without knowing such information, however, might result in misleading conclusions. Here, we have overcome the weakness of candidate gene-based methylation analyses by monitoring the leukocyte composition at the same time. This enabled us to uncover the underlying relationship, peculiar to pregnancy, between BMI and leukocyte proportion and their effects on the methylation of adiposity-related differentially methylated sites.

## 4. Materials and Methods

### 4.1. Study Population and Design

The **B**irth **C**ohort **G**ene and **EN**vironment **I**nteraction **S**tudy of **T**MDU (BC-GENIST) is a prospective mother–offspring cohort designed to evaluate the effects of the prenatal environment and genotype on the epigenetic state of mothers and their offspring; it is still collecting data at Tokyo Medical and Dental University, Medical Hospital, and has done so since November 2015. Initially, pregnant women aged 20 years and above were recruited for a prenatal checkup. Among the BC-GENIST participants, 52 pregnant women with singleton term pregnancies were enrolled in this study. In order to assess the effect of leukocyte composition on the methylation, we also collected blood samples from healthy non-pregnant women (*n* = 7). Written informed consent was obtained from all the participants. Our study was approved by the Institutional Review Board of the Medical Research Institute and Faculty of Medicine, Tokyo Medical and Dental University (No. G2000-181, 29 July 2014). The characteristics of the birth cohort participants, who were non-smokers, are shown in Table S1. Few women were diagnosed with gestational diabetes mellitus or thyroid disease, but those diseases were under control during the study period. Moreover, self-reported pre-pregnancy BMI and other data collected from medical records were used. The distribution of pre-pregnancy BMI ranges in our cohort (BMI < 18.5, 17%; 18.5 ≤ BMI < 25, 73%; 25 ≤ BMI, 10%) was similar to that found in the Japanese National Heath and Nutritional Survey at 2016 [31]. BMI data and blood samples were collected at two time points during prenatal visits: time point 1 (T1), intended to represent the second trimester from week 13 to week 29, and time point 2 (T2), to represent the third trimester from week 34 to week 37 of gestational age.

### 4.2. Maternal Blood Sample Collection and Serum LDL-C Quantification

Peripheral blood samples were collected from pregnant women using two collecting vials: one for the extraction of DNA (VP-NA070K containing EDTA; TERUMO, Tokyo, Japan) and the other for the serum separation (VP-AS109K50 containing fibrin; TERUMO). The DNA was extracted by an automated system to extract the nucleic acid (GENE PREP STAR NA-480; KURABO, Osaka, Japan), and the serum was separated by centrifugation. Since blood samples were not always collected during a fasting state, serum LDL-C was measured as a lipid parameter, which is considered unaffected by the diet condition. Serum LDL-C levels were determined by an enzymatic method in HOKEN KAGAKU KENKYUJO (HOKEN KAGAKU KENKYUJO, Yokohama, Japan).

### 4.3. Collection of Control Blood Samples for the Cell Fractionation and Cell Counts

Peripheral blood samples were collected from non-pregnant women, and the neutrophil, monocyte, and lymphocyte fractions were isolated using an EasySep™ Direct Human Neutrophil Isolation Kit, an EasySep™ Direct Human Monocyte Isolation Kit, and an EasySep™ Direct Human Total Lymphocyte Isolation Kit (STEMCELL Technologies, Vancouver, Canada), according to the manufacturer’s protocol. A part of the fractionated cells was subjected to Cytospin smears (Shandon Cytospin 3; Thermo Fisher Scientific, Grand Island, NY, USA). In addition, a whole blood smear was prepared for each sample. The leukocyte cell counts under the microscope were performed using a standard procedure by two trained doctors [32]. The purity of the cell separation was 98.8 ± 1.2% (neutrophils), 95.5 ± 3.3% (monocytes), and 99.7 ± 0.6% (lymphocytes). The DNA from non-pregnant women was isolated using a QIA-amp DNA Mini Kit (Qiagen, Hilden, Germany).

### 4.4. Methylation Analysis Using EpiTYPER

Methylation analysis using the MassARRAY EpiTYPER (Agena Bioscience, San Diego, CA, USA) was performed according to the standard protocol by Suchiman et al. [33]. In brief, DNA quality was examined using gel electrophoresis, and the concentration was quantified using a Quant-it PicoGreen dsDNA Assay Kit (Thermo Fisher Scientific, Tokyo, Japan). Bisulfite treatment was performed with 500 ng of extracted DNA using an EZ DNA Methylation Kit (Zymo Research, Irvine, CA, USA). Subsequently, PCR amplification was performed in triplicate with bisulfite-treated genomic DNA using primers for each genomic locus, which were designed using the web tool EpiDesigner (https://www.epidesigner.com/). All the amplicon information is given in Table S2. After T-cleavage reactions using a MassCLEAVE™ Reagent Kit (Agena Bioscience, San Diego, CA, USA), samples were transferred to a 384 SpectroCHIP and read by a mass spectrometer MassARRAY system (Agena Bioscience, San Diego, CA, USA). The DNA methylation levels for each CpG unit were calculated by EpiTYPER software v1.3 (Agena Bioscience). The data passed all the quality controls described in reference [33] and were used for the analyses in this study. 

### 4.5. Construction of a Standard Curve for Specific Cell Type Proportion vs. Marker Site Methylation

We extracted several neutrophil-specific methylation markers (such as cg22840076 in *HNRNPUL1*, cg21762728 in *ERMARD*, cg17385088 (intergenic, chr4:153611560-153611561) and cg23954655 in *STK24*) from the open source methylation reference datasets FlowSorted.Blood.450k [34] and FlowSorted.Blood.EPIC. [35]. Among them, we found that the methylation levels of cg23954655 (intron 1 CpG2 in our analysis) in serine/threonine kinase 24 gene (*STK24*), which is important for degranulation of neutrophils, were highly correlated with the neutrophil proportion measured by the white blood cell count (Figure S3). The formula was constructed by plotting the neutrophil proportion observed along the *x* axis and the DNA methylation levels of *STK24* CpG_2 along the *y* axis. 

### 4.6. Statistical Analysis

SPSS statistical software (version 24; IBM, Armonk, USA) was used for the statistical analyses. Statistical differences in variables between T1 and T2 were tested using paired *t*-tests. We performed a linear regression analysis with methylation levels (%) as the dependent variable and with the adiposity phenotype or the specific cell type proportion as the independent variable. The β coefficients with 95% confidential intervals and *p*-values were reported. Since this study was a confirmatory analysis of the adiposity–methylation association and a comparison of the adiposity effect between two timepoints, multiplicity adjustment was not performed, and statistical significance was assigned at *p* < 0.05. 

## 5. Conclusions

During mid-to-late gestation, maternal metabolism switches from an anabolic to a catabolic state, which is mainly characterized by an alteration in lipid metabolism. Although the methylation of *SREBF1* intron 1 was associated with BMI and LDL-C at mid-gestation, this association was attenuated at late gestation. Meanwhile, the BMI association with *CPT1A* intron 1 methylation was strengthened at late gestation, which was mediated by a BMI-dependent change in the lymphocyte proportion. Thus, this study has shown that leukocyte methylation of the adiposity-related differentially methylated regions is sensitive to the metabolic and immunological changes that occur during mid-to-late gestation.

## Figures and Tables

**Table 1:** Comparison between T1 and T2 methylation levels of *CPT1A* and *SREBF1* variably methylated sites (*n* = 52).

Gene	Genomic Context	EpiTYPER CpG Unit	Mean Methylation (%) (SD)		*p*-Value *
			T1	T2	
*CPT1A*	Intron 1	CpG_2 ^a^	1.9 (0.7)	1.9 (0.6)	8.2 × 10^−1^
		CpG_3	8.9 (1.3)	9.1 (1.0)	2.4 × 10^−1^
		CpG_4	4.5 (1.1)	4.7 (0.9)	7.3 × 10^−2^
		CpG_6	11.9 (3.8)	12.5 (4.0)	2.8 × 10^−1^
		CpG_7	4.8 (1.3)	4.9 (1.1)	3.6 × 10^−1^
		CpG_8 ^b^	6.5 (1.5)	6.8 (1.3)	1.4 × 10^−1^
*SREBF1*	Intron 1	CpG_1	46.6 (2.8)	47.2 (2.9)	5.0 × 10^−3^
		CpG_3	36.9 (4.0)	36.7 (4.2)	7.5 × 10^−1^
		CpG_4 ^c^	71.3 (2.5)	72.7 (2.3)	1.6 × 10^−7^
*CPT1A*	Exon 15	CpG_2.3 ^d^	26.0 (13.3)	29.1 (13.0)	2.9 × 10^−5^
		CpG_5.6	48.5 (10.5)	52.0 (10.1)	2.3 × 10^−6^
		CpG_8.9	73.5 (5.7)	75.2 (5.7)	3.0 × 10^−4^
		CpG_10	38.7 (10.3)	41.7 (9.9)	3.3 × 10^−5^
		CpG_12	19.5 (5.8)	21.9 (5.6)	6.4 × 10^−4^

Values given are mean (SD) of the methylation in percentage. a—cg00574958, b—cg17058475, c—is a proxy for cg11024682, d—cg14249520. * *p* value for the difference between T1 and T2 methylation levels analyzed using a paired *t*-test. The correlation matrix for the methylation of all the CpGs within *CPT1A* intron 1, *SREBF1* intron 1, and *CPT1A* exon 15 is shown in Table S3.

## Supplementary Materials

Supplementary materials can be found at https://www.mdpi.com/1422-0067/20/5/1066/s1. Table S1: Characteristics of pregnant women included in this study (*N* = 52); Table S2: Amplicon information; Table S3: Methylation correlation matrix; Figure S1: Pre-pregnancy BMI and gestational BMI at T1 and T2; Figure S2: Association of LDL-C and methylation levels at mid- and late gestation; Figure S3: Construction of a reference curve for the relationship between observed neutrophil proportion (%) and methylation levels at *STK24*_CpG2 (cg23954655); Figure S4: Correlation between estimations of neutrophil proportion change derived from two different neutrophil markers; Figure S5: The relationship between observed lymphocyte proportion (%) and methylation levels of *CPT1A* intron 1.

## Abbreviations

CpG	5′-Cytosine-phosphate-guanine-3′
EWAS	Epigenome-wide association study
*CPT1A*	Carnitine palmitoyltransferase 1A
*SREBF1*	Sterol regulatory element-binding transcription factor 1
*STK24*	Serine/threonine kinase 24
BMI	Body mass index
LDL-C	Low-density lipoprotein cholesterol
BC-GENIST	Birth Cohort Gene and ENvironment Interaction Study of TMDU
