# Supplementary material for: Metabolic and Immunological Shifts during Mid-to-Late Gestation Influence Maternal Blood Methylation of CPT1A and SREBF1
Source: Int J Mol Sci. 2019 Mar 1;20(5):1066. doi: 10.3390/ijms20051066 (PMC6429071; doi:10.3390/ijms20051066)
Supplement: Supplementary file 1 [file ijms-20-01066-s001.zip › ijms-451081-supplementary.pptx]

## Slide 1
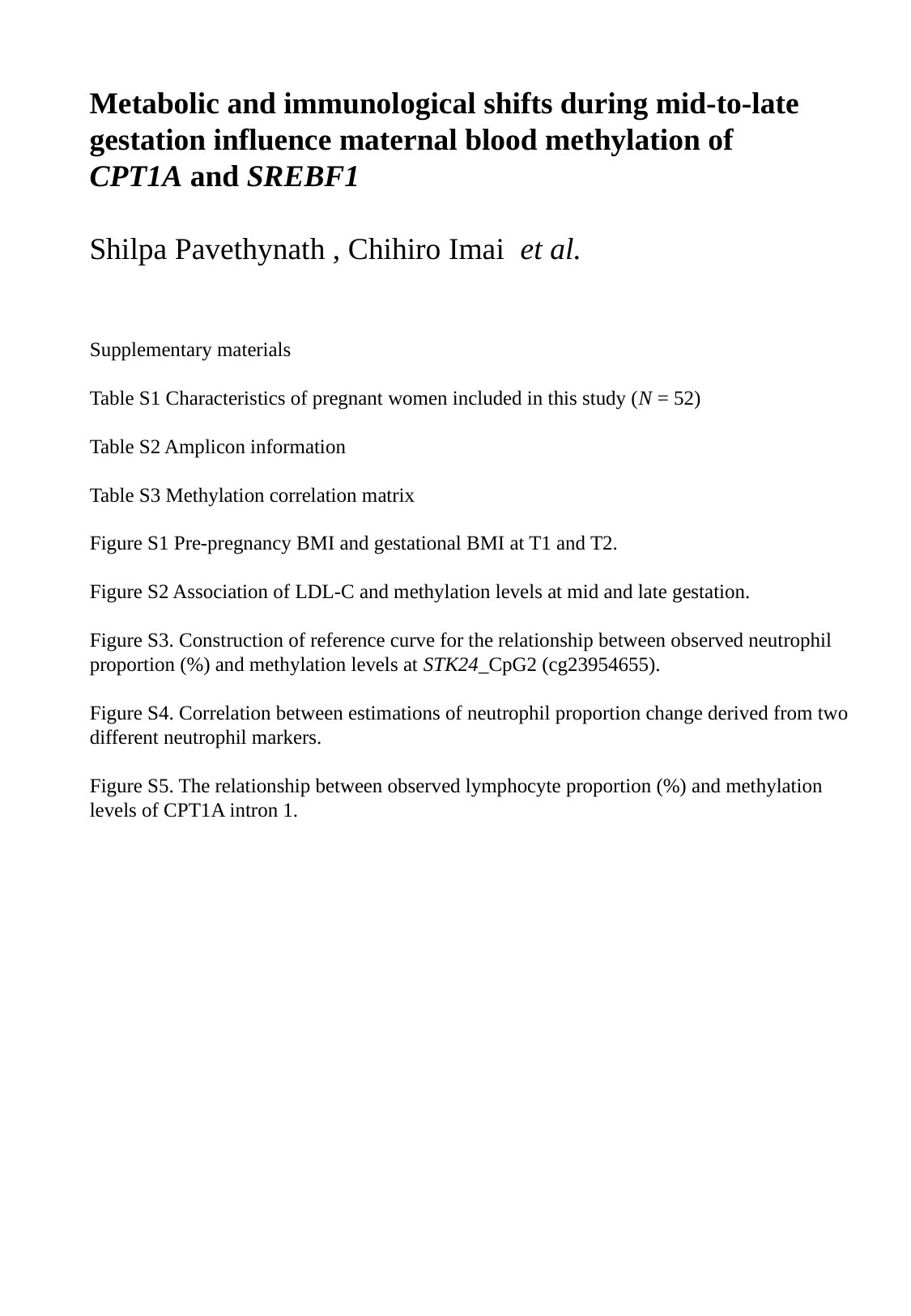

Metabolic and immunological shifts during mid-to-late gestation influence maternal blood methylation of CPT1A and SREBF1
Shilpa Pavethynath , Chihiro Imai et al.
Supplementary materials
Table S1 Characteristics of pregnant women included in this study (N = 52)
Table S2 Amplicon information
Table S3 Methylation correlation matrix
Figure S1 Pre-pregnancy BMI and gestational BMI at T1 and T2.
Figure S2 Association of LDL-C and methylation levels at mid and late gestation.
Figure S3. Construction of reference curve for the relationship between observed neutrophil proportion (%) and methylation levels at STK24_CpG2 (cg23954655).
Figure S4. Correlation between estimations of neutrophil proportion change derived from two different neutrophil markers.
Figure S5. The relationship between observed lymphocyte proportion (%) and methylation levels of CPT1A intron 1.

## Slide 2
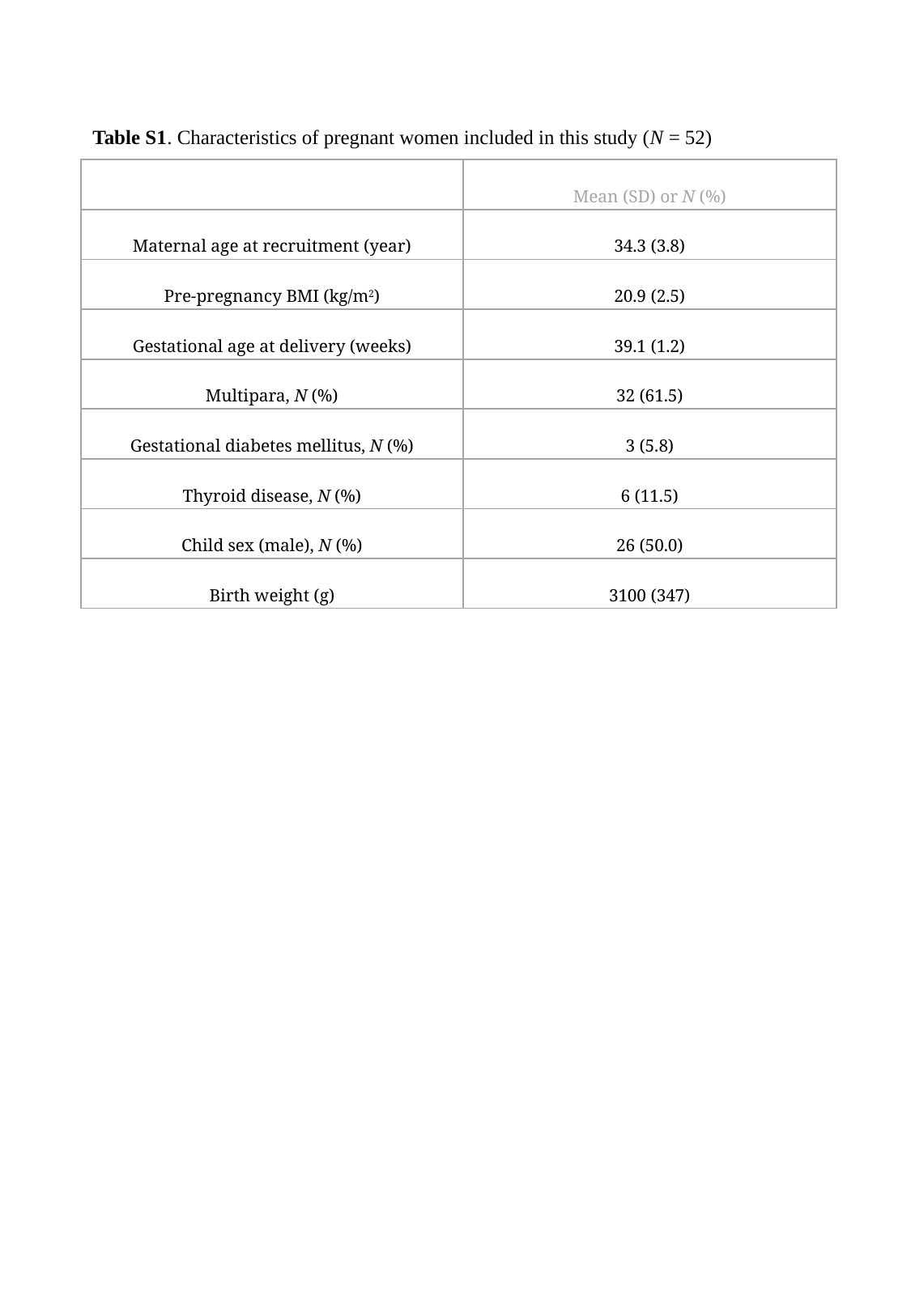

Table S1. Characteristics of pregnant women included in this study (N = 52)
| | Mean (SD) or N (%) |
| --- | --- |
| Maternal age at recruitment (year) | 34.3 (3.8) |
| Pre-pregnancy BMI (kg/m2) | 20.9 (2.5) |
| Gestational age at delivery (weeks) | 39.1 (1.2) |
| Multipara, N (%) | 32 (61.5) |
| Gestational diabetes mellitus, N (%) | 3 (5.8) |
| Thyroid disease, N (%) | 6 (11.5) |
| Child sex (male), N (%) | 26 (50.0) |
| Birth weight (g) | 3100 (347) |

## Slide 3
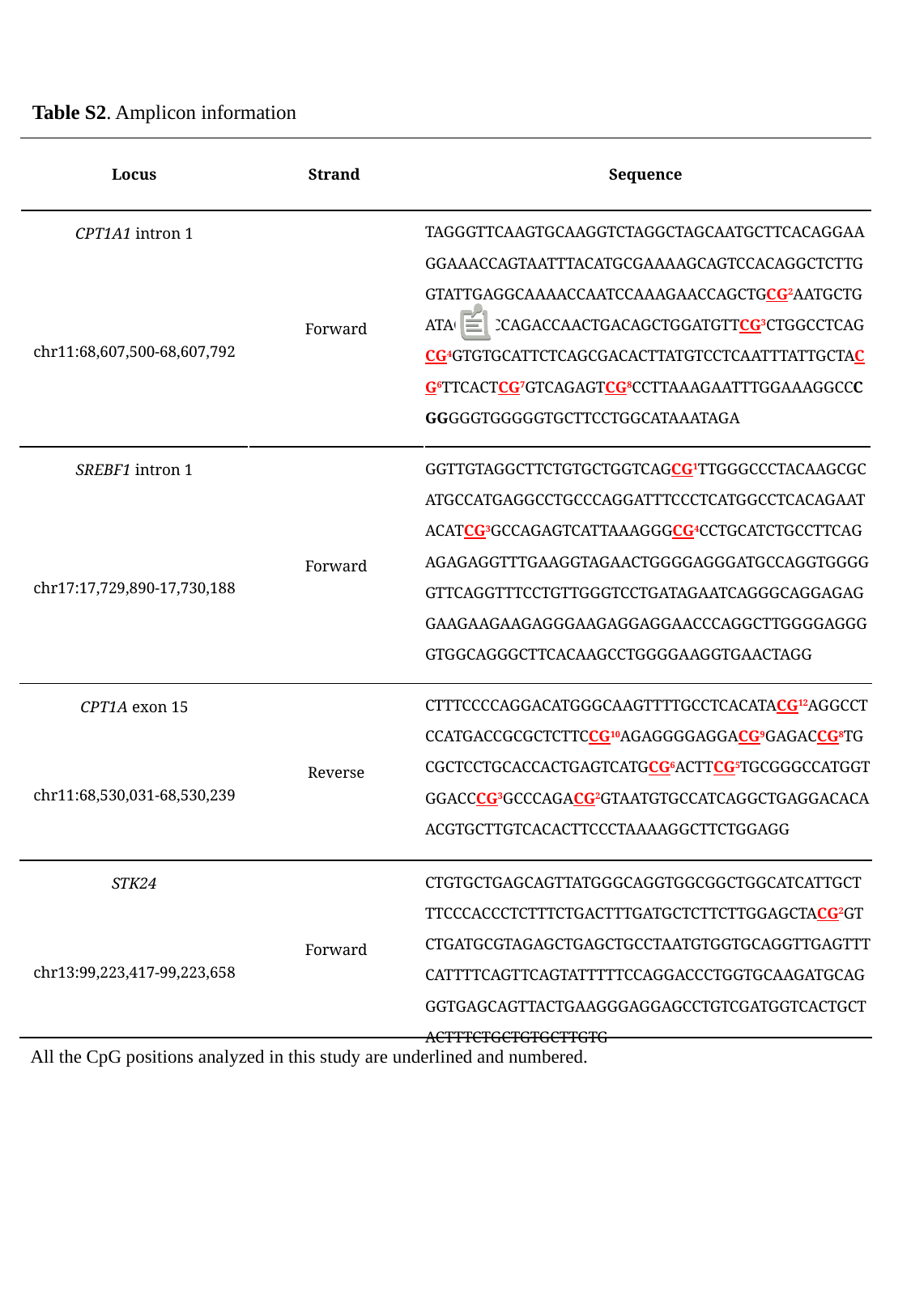

Table S2. Amplicon information
| Locus | Strand | Sequence |
| --- | --- | --- |
| CPT1A1 intron 1 | Forward | TAGGGTTCAAGTGCAAGGTCTAGGCTAGCAATGCTTCACAGGAAGGAAACCAGTAATTTACATGCGAAAAGCAGTCCACAGGCTCTTGGTATTGAGGCAAAACCAATCCAAAGAACCAGCTGCG2AATGCTGATAGTCCCCAGACCAACTGACAGCTGGATGTTCG3CTGGCCTCAGCG4GTGTGCATTCTCAGCGACACTTATGTCCTCAATTTATTGCTACG6TTCACTCG7GTCAGAGTCG8CCTTAAAGAATTTGGAAAGGCCCGGGGGTGGGGGTGCTTCCTGGCATAAATAGA |
| chr11:68,607,500-68,607,792 | | |
| SREBF1 intron 1 | Forward | GGTTGTAGGCTTCTGTGCTGGTCAGCG1TTGGGCCCTACAAGCGCATGCCATGAGGCCTGCCCAGGATTTCCCTCATGGCCTCACAGAATACATCG3GCCAGAGTCATTAAAGGGCG4CCTGCATCTGCCTTCAGAGAGAGGTTTGAAGGTAGAACTGGGGAGGGATGCCAGGTGGGGGTTCAGGTTTCCTGTTGGGTCCTGATAGAATCAGGGCAGGAGAGGAAGAAGAAGAGGGAAGAGGAGGAACCCAGGCTTGGGGAGGGGTGGCAGGGCTTCACAAGCCTGGGGAAGGTGAACTAGG |
| chr17:17,729,890-17,730,188 | | |
| CPT1A exon 15 | Reverse | CTTTCCCCAGGACATGGGCAAGTTTTGCCTCACATACG12AGGCCTCCATGACCGCGCTCTTCCG10AGAGGGGAGGACG9GAGACCG8TGCGCTCCTGCACCACTGAGTCATGCG6ACTTCG5TGCGGGCCATGGTGGACCCG3GCCCAGACG2GTAATGTGCCATCAGGCTGAGGACACAACGTGCTTGTCACACTTCCCTAAAAGGCTTCTGGAGG |
| chr11:68,530,031-68,530,239 | | |
| STK24 | Forward | CTGTGCTGAGCAGTTATGGGCAGGTGGCGGCTGGCATCATTGCTTTCCCACCCTCTTTCTGACTTTGATGCTCTTCTTGGAGCTACG2GTCTGATGCGTAGAGCTGAGCTGCCTAATGTGGTGCAGGTTGAGTTTCATTTTCAGTTCAGTATTTTTCCAGGACCCTGGTGCAAGATGCAGGGTGAGCAGTTACTGAAGGGAGGAGCCTGTCGATGGTCACTGCTACTTTCTGCTGTGCTTGTG |
| chr13:99,223,417-99,223,658 | | |
All the CpG positions analyzed in this study are underlined and numbered.

## Slide 4
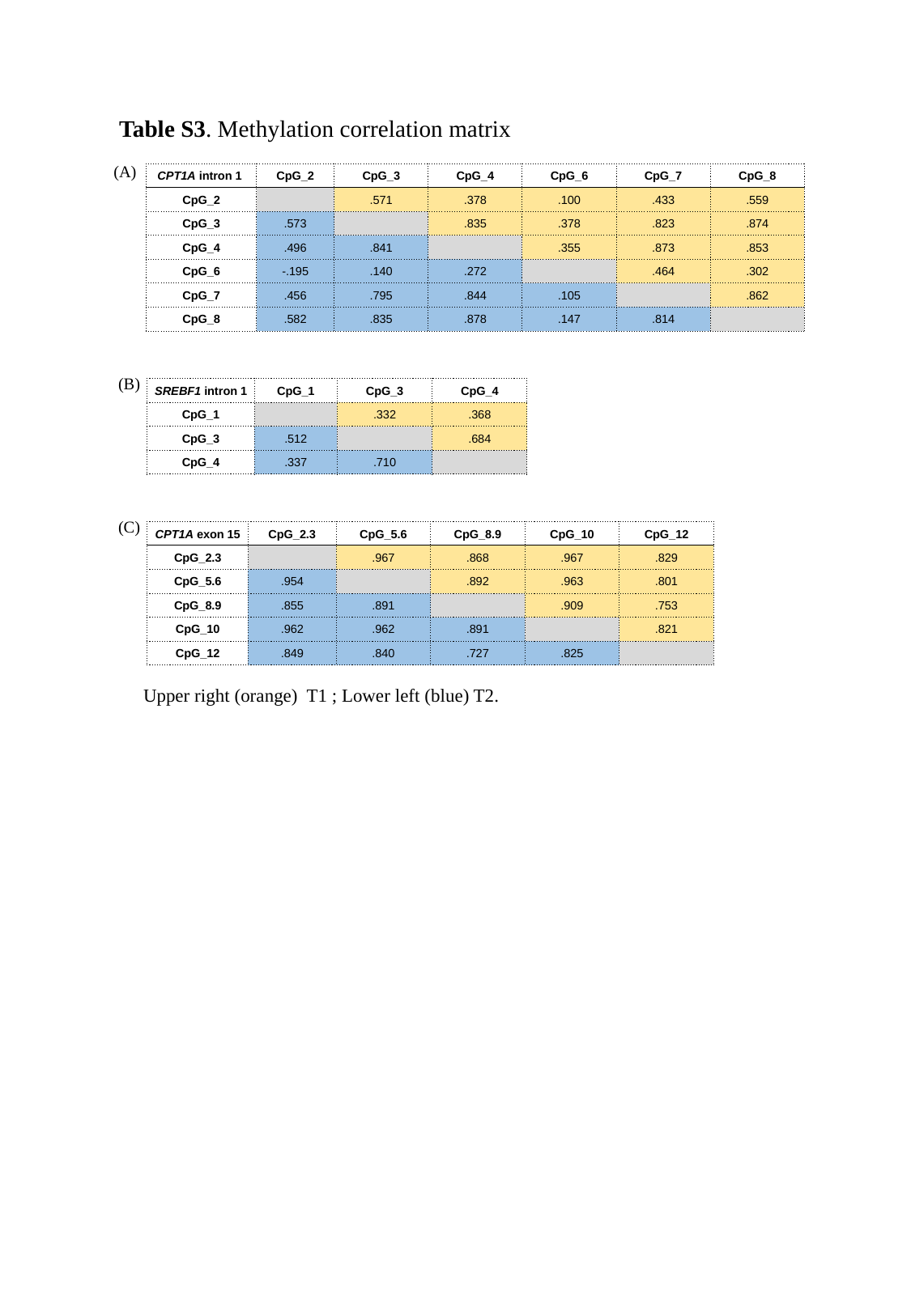

Table S3. Methylation correlation matrix
(A)
| CPT1A intron 1 | CpG\_2 | CpG\_3 | CpG\_4 | CpG\_6 | CpG\_7 | CpG\_8 |
| --- | --- | --- | --- | --- | --- | --- |
| CpG\_2 | | .571 | .378 | .100 | .433 | .559 |
| CpG\_3 | .573 | | .835 | .378 | .823 | .874 |
| CpG\_4 | .496 | .841 | | .355 | .873 | .853 |
| CpG\_6 | -.195 | .140 | .272 | | .464 | .302 |
| CpG\_7 | .456 | .795 | .844 | .105 | | .862 |
| CpG\_8 | .582 | .835 | .878 | .147 | .814 | |
(B)
| SREBF1 intron 1 | CpG\_1 | CpG\_3 | CpG\_4 |
| --- | --- | --- | --- |
| CpG\_1 | | .332 | .368 |
| CpG\_3 | .512 | | .684 |
| CpG\_4 | .337 | .710 | |
(C)
| CPT1A exon 15 | CpG\_2.3 | CpG\_5.6 | CpG\_8.9 | CpG\_10 | CpG\_12 |
| --- | --- | --- | --- | --- | --- |
| CpG\_2.3 | | .967 | .868 | .967 | .829 |
| CpG\_5.6 | .954 | | .892 | .963 | .801 |
| CpG\_8.9 | .855 | .891 | | .909 | .753 |
| CpG\_10 | .962 | .962 | .891 | | .821 |
| CpG\_12 | .849 | .840 | .727 | .825 | |
Upper right (orange) T1 ; Lower left (blue) T2.

## Slide 5
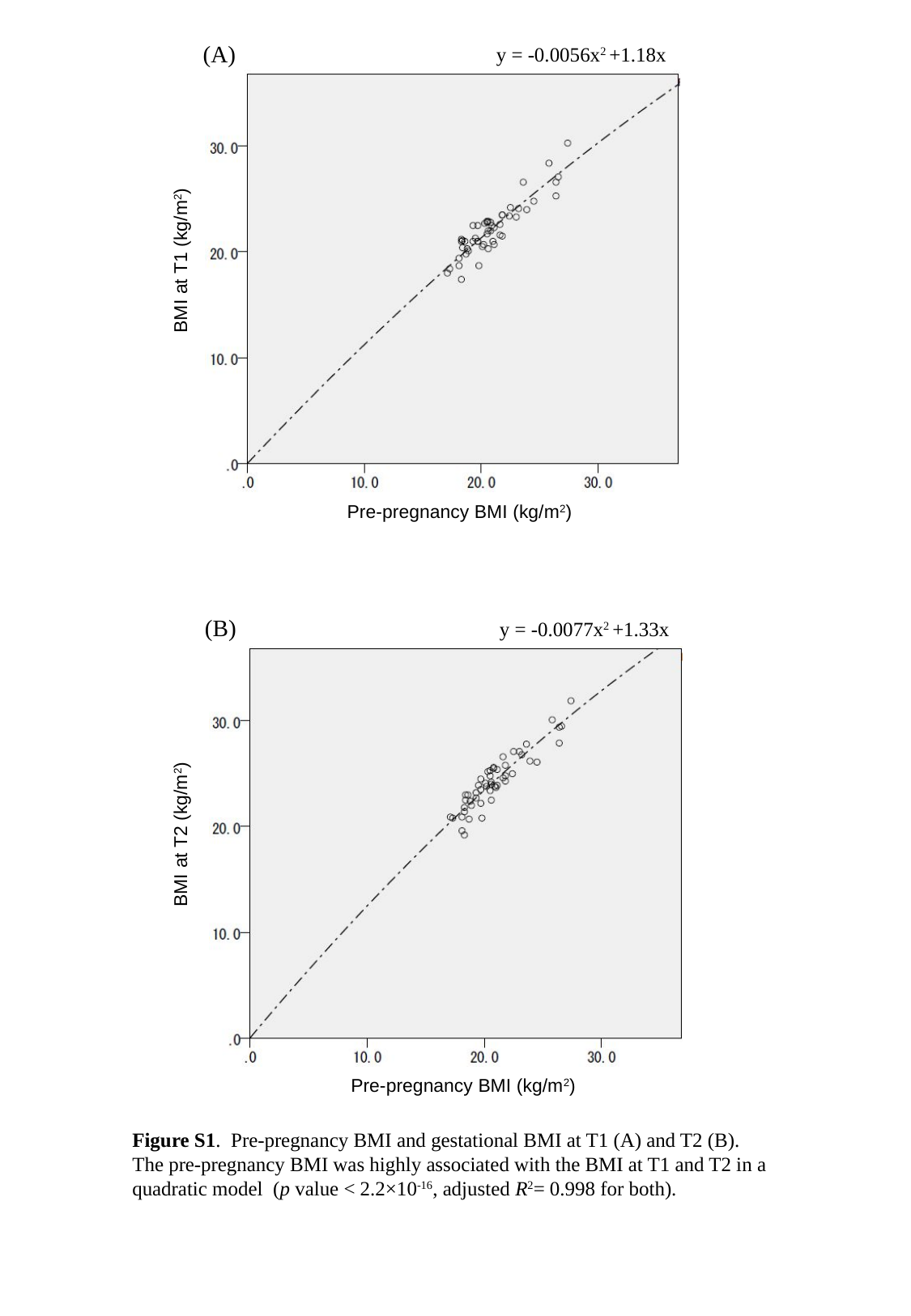

(A)
y = -0.0056x2 +1.18x
BMI at T1 (kg/m2)
Pre-pregnancy BMI (kg/m2)
(B)
y = -0.0077x2 +1.33x
BMI at T2 (kg/m2)
Pre-pregnancy BMI (kg/m2)
Figure S1. Pre-pregnancy BMI and gestational BMI at T1 (A) and T2 (B).
The pre-pregnancy BMI was highly associated with the BMI at T1 and T2 in a quadratic model (p value < 2.2×10^-16^, adjusted R2= 0.998 for both).

## Slide 6
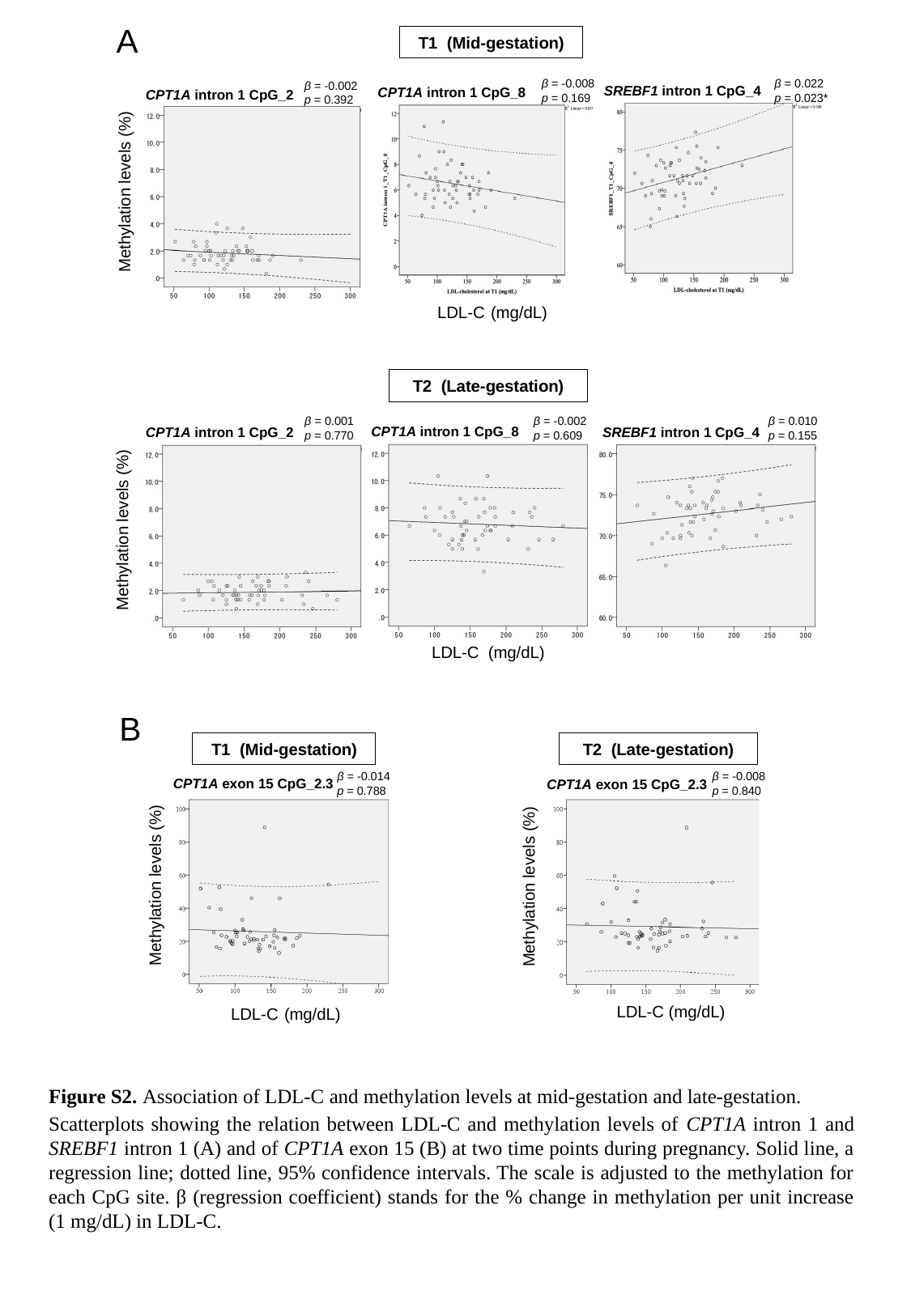

A
T1 (Mid-gestation)
β = -0.008
p = 0.169
β = 0.022
p = 0.023*
β = -0.002
p = 0.392
 SREBF1 intron 1 CpG_4
 CPT1A intron 1 CpG_8
CPT1A intron 1 CpG_2
Methylation levels (%)
LDL-C (mg/dL)
T2 (Late-gestation)
β = 0.001
p = 0.770
β = -0.002
p = 0.609
β = 0.010
p = 0.155
 CPT1A intron 1 CpG_8
 SREBF1 intron 1 CpG_4
CPT1A intron 1 CpG_2
Methylation levels (%)
LDL-C (mg/dL)
B
T1 (Mid-gestation)
T2 (Late-gestation)
β = -0.014
p = 0.788
β = -0.008
p = 0.840
CPT1A exon 15 CpG_2.3
CPT1A exon 15 CpG_2.3
Methylation levels (%)
Methylation levels (%)
LDL-C (mg/dL)
LDL-C (mg/dL)
Figure S2. Association of LDL-C and methylation levels at mid-gestation and late-gestation.
Scatterplots showing the relation between LDL-C and methylation levels of CPT1A intron 1 and SREBF1 intron 1 (A) and of CPT1A exon 15 (B) at two time points during pregnancy. Solid line, a regression line; dotted line, 95% confidence intervals. The scale is adjusted to the methylation for each CpG site. β (regression coefficient) stands for the % change in methylation per unit increase (1 mg/dL) in LDL-C.

## Slide 7
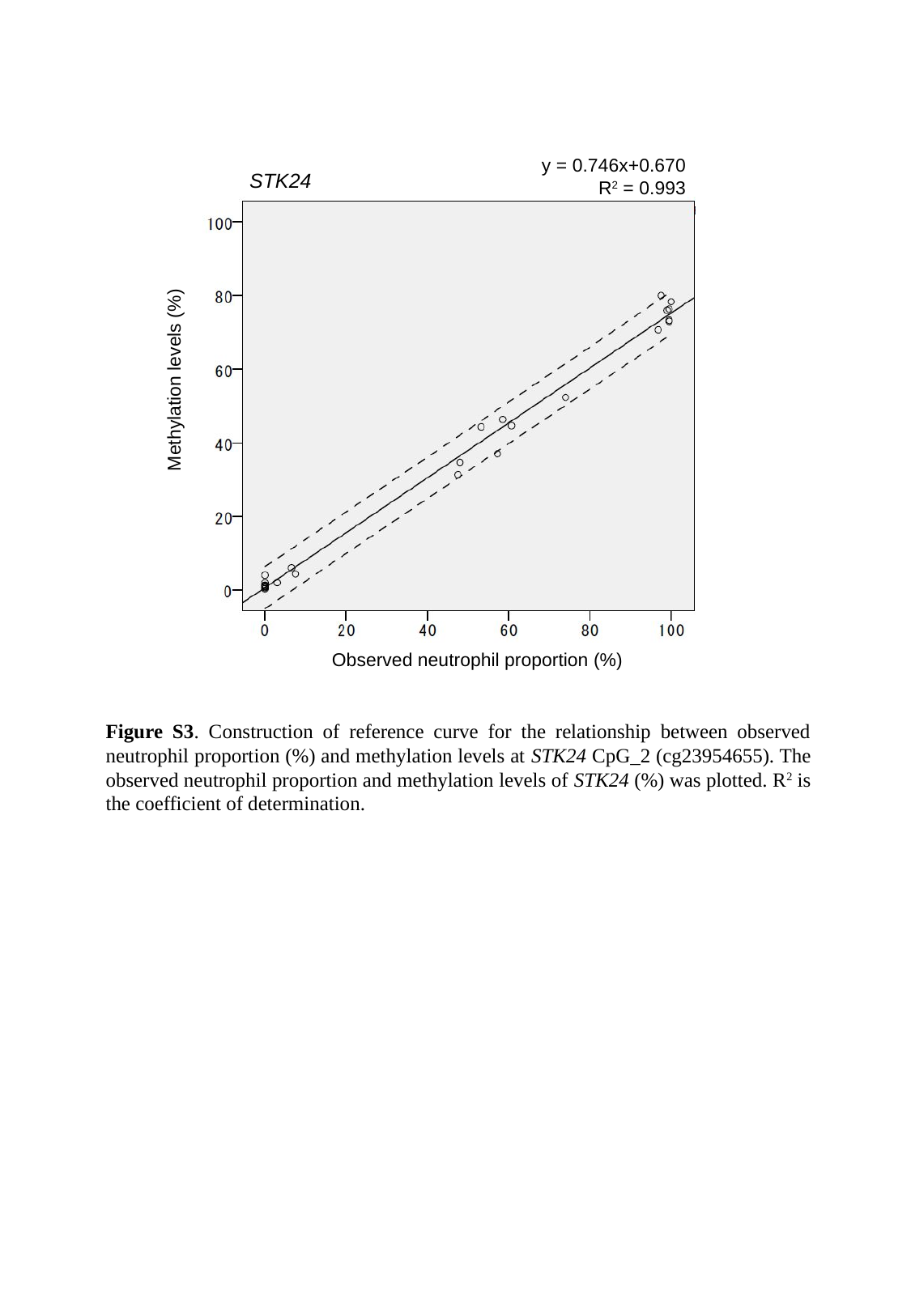

y = 0.746x+0.670
 R2 = 0.993
STK24
Methylation levels (%)
Observed neutrophil proportion (%)
Figure S3. Construction of reference curve for the relationship between observed neutrophil proportion (%) and methylation levels at STK24 CpG_2 (cg23954655). The observed neutrophil proportion and methylation levels of STK24 (%) was plotted. R2 is the coefficient of determination.

## Slide 8
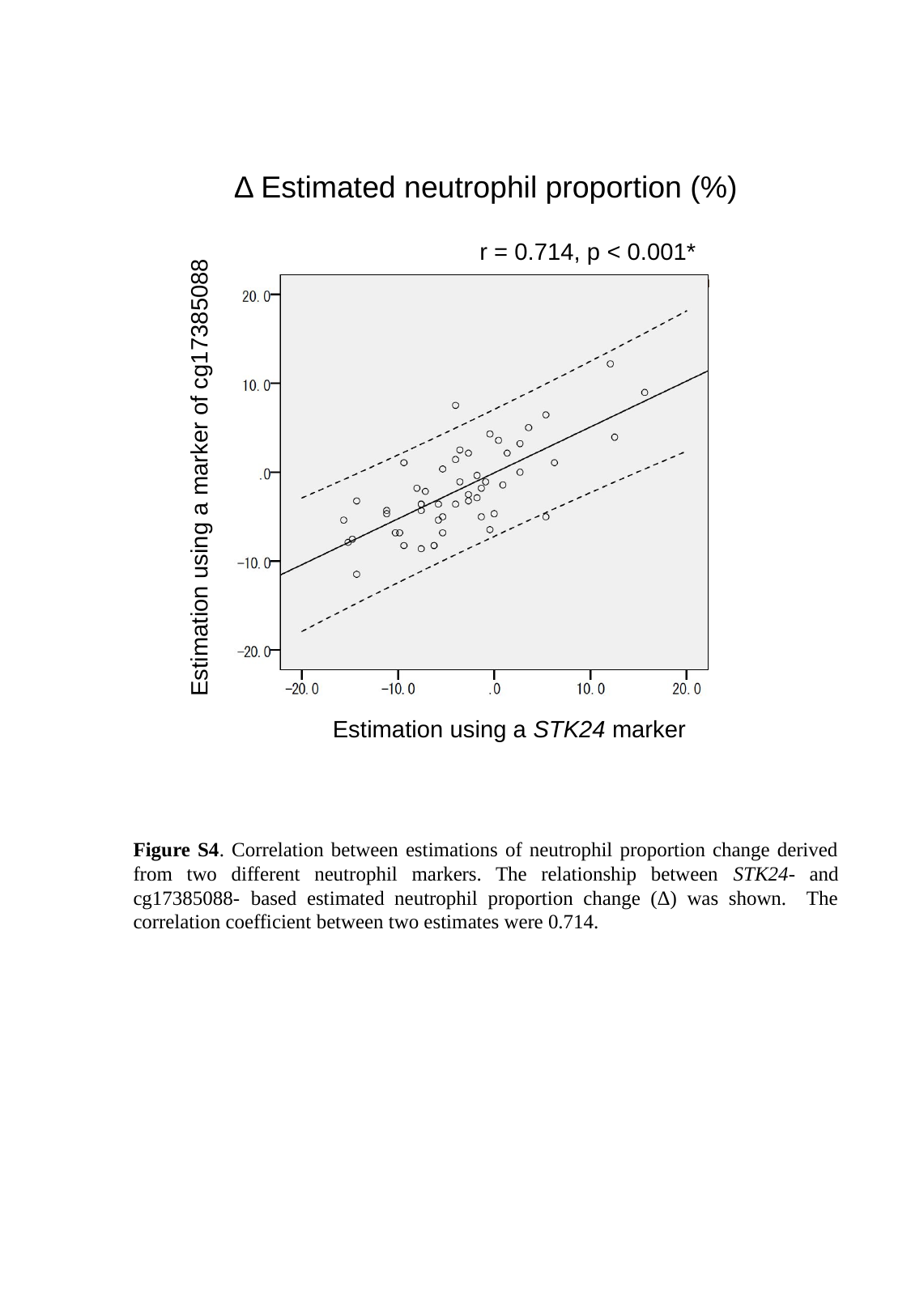

Δ Estimated neutrophil proportion (%)
r = 0.714, p < 0.001*
Estimation using a marker of cg17385088
Estimation using a STK24 marker
Figure S4. Correlation between estimations of neutrophil proportion change derived from two different neutrophil markers. The relationship between STK24- and cg17385088- based estimated neutrophil proportion change (Δ) was shown. The correlation coefficient between two estimates were 0.714.

## Slide 9
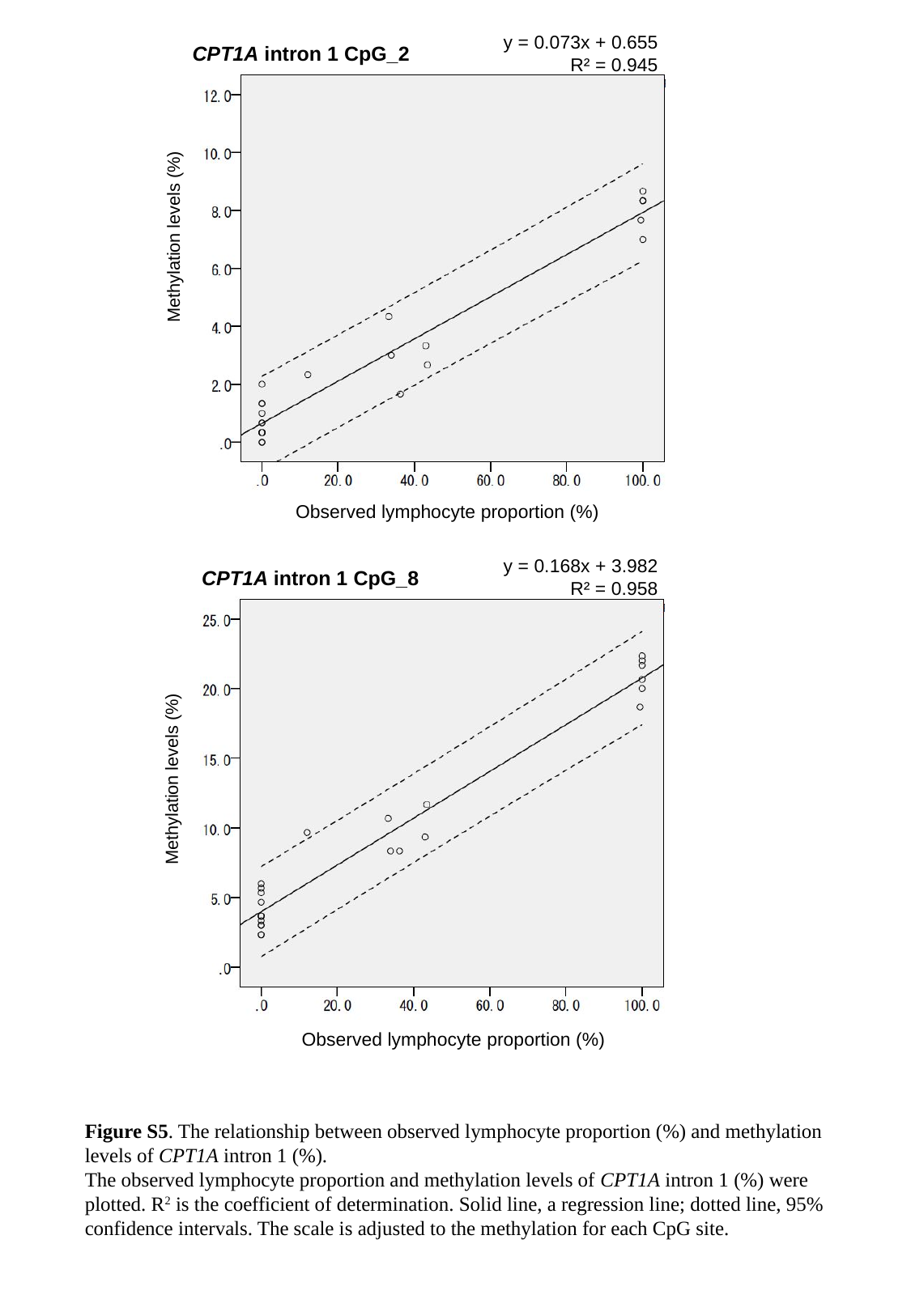

y = 0.073x + 0.655
 R² = 0.945
CPT1A intron 1 CpG_2
Methylation levels (%)
Observed lymphocyte proportion (%)
y = 0.168x + 3.982
 R² = 0.958
CPT1A intron 1 CpG_8
Methylation levels (%)
Observed lymphocyte proportion (%)
Figure S5. The relationship between observed lymphocyte proportion (%) and methylation levels of CPT1A intron 1 (%).
The observed lymphocyte proportion and methylation levels of CPT1A intron 1 (%) were plotted. R2 is the coefficient of determination. Solid line, a regression line; dotted line, 95% confidence intervals. The scale is adjusted to the methylation for each CpG site.
